# Successful Treatment of Auricular Dystonia by Unilateral Pallidothalamic Tractotomy

**DOI:** 10.5334/tohm.579

**Published:** 2021-01-22

**Authors:** Kilsoo Kim, Shiro Horisawa, Kotaro Kohara, Taku Nonaka, Takakazu Kawamata, Takaomi Taira

**Affiliations:** 1 Department of Neurosurgery, Neurological Institute, Tokyo Women’s Medical University, Tokyo, Japan

**Keywords:** ear dyskinesia, auricular dystonia, pallidothalamic tract

## Abstract

**Background:**

Ear movement disorders are rarely reported. Although some patients may respond to botulinum toxin injections, reports on surgical treatment options remain limited.

**Case Report:**

A 57-year-old woman was diagnosed with auricular dystonia, which was refractory to botulinum toxin injections. Since involuntary movement and pain were predominantly present on the right side and the patient rejected the implantation of a mechanical device, we decided to perform left pallidothalamic tractotomy. Immediately following lesioning, bilateral ear movements and right auricular pain ceased with no complications.

**Discussion:**

Stereotactic neurosurgical treatment can be an alternative for auricular dystonia.

**Highlights:**

Ear movement disorders, such as auricular myoclonus or dystonia, are rarely reported.

The present case was refractory to repetitive botulinum toxin injections and oral medications.

To the best of our knowledge, this is the first case of auricular dystonia that successfully improved with stereotactic neurosurgical treatment (pallidothalamic tractotomy).

## INTRODUCTION

Moving ears syndrome (ear movement disorders), including auricular myoclonus, dystonia or focal motor seizure, are rarely reported [1,2,3,4,5,6]. Three major muscles located around the auricle, namely the anterior, superior, and posterior auricular muscles, are functionally vestigial in humans. Previous studies have shown that ear movement disorders can successfully be treated with botulinum toxin injections [2,5,7]; however, to date, there have been no reports on surgical treatment. Herein, we report a patient with auricular dystonia, which was refractory to botulinum toxin injections, who was successfully treated with unilateral pallidothalamic tractotomy.

## CASE DESCRIPTION

The patient, a 57-year-old woman, had no significant history of traumatic injuries or psychiatric or movement disorders. Involuntary movement of the right ear had gradually developed at 53 years of age, and involuntary movement of the left ear had developed two months later. Initially, the movements were intermittent; bilateral ear movements had developed gradually, and became continuous with significant pain in the right auricle and posterior neck. She was diagnosed with focal auricular dystonia and received oral medicines including clonazepam (3 mg), pain medications (NSAID and tramadol hydrochloride), repetitive botulinum toxin injections (5–10 IU) into the right posterior auricular muscles, and occipital nerve blockers (lidocaine). However, these treatments were not effective in reducing pain and involuntary movements of the ear. She discontinued anticholinergic drugs due to side effects including dry mouth, sleepiness, and dizziness. Although botulinum toxin injections into the anterior auricular and/or superior muscle were partially effective for involuntary movement of the right auricle, she discontinued botulinum toxin injections due to high associated costs and unpredictable effects. She was ultimately referred to our hospital for surgical treatment evaluation. Her ear movements were predominantly on the right side. Involuntary muscle contractions were confirmed in the right anterior and superior auricular muscles of the right auricle (***Video 1***). In the left auricle, the main involuntary muscle contraction was confirmed to be in the superior auricular muscle (***Video 1***). She exhibited no voluntary control over these ear movements, and could not suppress them; significant pain was only present in the right auricle. There were no synchronous and rhythmic movements between the two ears. Involuntary movements were not confirmed during sleep and were mild in the morning. Her brain MRI showed no abnormalities; electromyography was not performed. Before the onset of the auricular movements, she had not taken any oral medications. We diagnosed her with focal auricular dystonia. Although we suggested deep brain stimulation, the patient rejected the implantation of a mechanical device. Since involuntary movements and pain were predominantly present on the right side, we decided to perform left pallidothalamic tractotomy after the patient provided written informed consent for the surgery.

**Video 1:** Preoperative condition.

**Figure 1:**
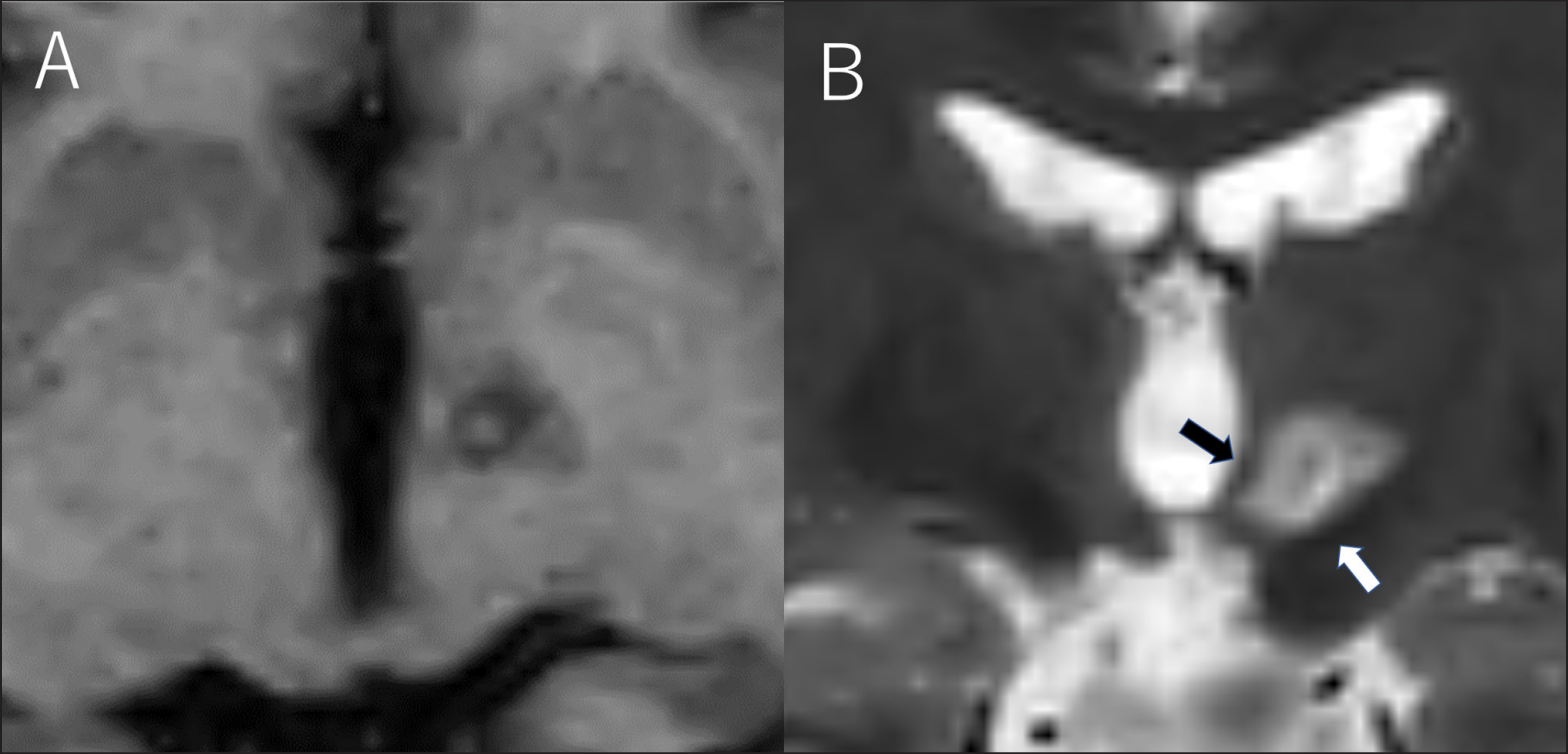
Postoperative MRI. T1-axial **(A)** and T2-coronal **(B)** MRI showing a lesion on the left pallidothalamic tract. Black arrow showing the mammillothalamic tract and white arrow showing the subthalamic nucleus.

T2-weighted brain MRI was used to identify a potential surgical target, while brain lab elements were used for stereotactic planning. The target was 9 mm lateral, 0.5 mm posterior, and 3.5 mm inferior to the midpoint of the anterior and posterior commissures. The surgery was performed under local anesthesia. A Leksell neurogenerator and monopolar radiofrequency electrode (1 mm diameter, 4 mm insulated tip) were used to perform macrostimulation and lesioning. Macrostimulation at the target (100 µs, 133 Hz, and 1–3.5 mA) induced no abnormal sensory or motor responses. We made two contiguous lesions and a 3 mm withdrawn point at 70ºC/40 seconds. Immediately following lesioning (on the day of the surgery), bilateral ear movements had ceased (***Video 2***). The right auricular pain was completely resolved, while that in the posterior neck was not. Post-operative brain MRI showed a coagulated lesion located on the intended target (***Figure 1***). There were no significant complications, although the patient continued to complain of discomfort in the left auricle despite no visual observations of involuntary movements 3-months postoperatively.

**Video 2:** Postoperative condition.

## DISCUSSION

To the best of our knowledge, this is the first reported case of auricular dystonia (moving ears syndrome) successfully improved with stereotactic neurosurgical intervention. The present case was refractory to repetitive botulinum toxin injections and oral medications, including clonazepam. The significant pain in the right auricle and the involuntary movements were disabling; therefore, we decided to perform stereotactic neurosurgical intervention.

Moving ears syndrome has rarely been reported; it is associated with involuntary muscle contractions in the superior, anterior, and posterior auricular muscles. Caviness et al. reported four cases of ear dyskinesia following carbamazepine treatment [1]. Chaudhuri also reported on two cases of focal ear dystonia treated by clonazepam and botulinum toxin injections into the superior and posterior auricular muscles [2]. Carluer et al. reported a single case of ear dyskinesia treated by botulinum toxin injections into the superior auricular muscles [5]. Alonso-Navarro et al. reported on a case of ear dystonia where they confirmed right posterior auricular irregular activity on a needle electromyogram with muscle activity lasting a few seconds; this was successfully treated with botulinum toxin injections [3]. However, the present case was refractory to repetitive botulinum toxin injections. For patients who show no response to botulinum toxin injections during focal dystonia treatment, stereotactic neurosurgical treatment can be an alternative option. The most widely available surgical treatment is deep brain stimulation (DBS) of the globus pallidus internus (GPi) [8]. Previous studies have reported significant efficacy of GPi-DBS for dystonia in the craniocervical region [9,10]. However, there have been no reports on surgical treatment for focal auricular dystonia. The primary complaints in the present case included involuntary movement and pain in the right auricle. Therefore, we believed that surgery on the left side was sufficient for treating the right auricular involuntary movement and pain. Our patient rejected DBS since it involves implantation of a mechanical device in the body; thus, we suggested unilateral lesioning surgery as a viable alternative treatment option.

Comorbid pain associated with moving ears syndrome has not yet been reported. In this case, the patient complained of right whole auricular pain, which developed after the onset of the right auricular involuntary movement. Pain reduction was achieved in concurrence with the improvement of right auricular involuntary movement after surgery. Sensory innervations of the auricle include the great auricular nerve for the postero-inferior region, lesser occipital nerve for the supero-posterior region, auricular branch of the vagus nerve for the anterior region, and the auricular temporal nerve (branch of the mandibular nerve) for the antero-superior region [11]. Involuntary auricular muscle movements may compress or entrap those nerves, probably resulting in comorbid auricular pain.

Pallidotomy is the most widely available lesioning surgery for dystonia [12,13], and we recently reported on the efficacy of lesioning on the pallidothalamic tract for dystonia [14]. In our experience, the effects of pallidothalamic tractotomy on dystonia are similar to those of pallidotomy (more than 150 cases). The GPi has several adjacent vital structures, including the internal capsule, optic tract, and the globus pallidus externus. Unexpected hemorrhage or lesioning dislocation on adjacent structures can induce hemiparesis, visual field disturbance, or parkinsonism. The pallidothalamic tract is located lateral to the mammillothalamic tract and above the subthalamic nucleus. Possible off-target complications include dyskinesia with ablation of the subthalamic nucleus and amnesia with ablation of the mammillothalamic tract. The subthalamic nucleus and mammillothalamic tract are clearly visualized as low-intensity areas on T2-MRI; both can be avoided by stereotactic planning. Therefore, we preferred the pallidothalamic tract over the GPi in the treatment of dystonia due to the relative ease of access.

Other than pallidothalamic tractotomy, available surgical treatments for the present case included DBS, focus ultrasound ablation, and peripheral denervation. DBS can provide significant improvement in craniocervical dystonia without irreversible lesions [9,10]. Dystonia in the craniocervical region including blepharospasm, tongue protrusion dystonia, or cervical dystonia usually require bilateral surgical interventions. However, previous studies reported on some patients with craniocervical dystonia who responded successfully to unilateral surgical intervention [15,16,17,18,19]. Bilateral ablative procedures are associated with risks of possible complications including dysarthria, dysphagia, and dysphonia. In our previous study, unilateral pallidotomy combined with contralateral pallidothalamic tractotomy for dystonia induced mild hypophonia in 6 of 11 patients [14]. Recurrence or deterioration of dystonia may develop in the present case at a later stage. Considering the possible complications and long-term effects, DBS is a safer surgical treatment compared to ablative treatments. Focused ultrasound pallidothalamic tractotomy, which allows for ablative lesions without an incision, is a safe and effective procedure for Parkinson’s disease [20]. However, bilateral pallidothalamic tractotomy using focused ultrasound also induced hypophonia [20]. The possible risks of bilateral focused ultrasound ablations may be same as those of bilateral radiofrequency ablations. Auricular muscles are innervated by the branches of the facial nerve [21]. Peripheral denervation of those branches can improve moving ears syndrome, which has not yet been reported.
